# Five-Day Changes in Biomarkers of Exposure Among Adult Smokers After Completely Switching From Combustible Cigarettes to a Nicotine-Salt Pod System

**DOI:** 10.1093/ntr/ntz206

**Published:** 2019-11-05

**Authors:** Joanna Jay, Erika L Pfaunmiller, Norman J Huang, Gal Cohen, Donald W Graff

**Affiliations:** 1 JUUL Labs, Inc., San Francisco, CA; 2 Celerion, Inc., Lincoln, NE

## Abstract

**Introduction:**

This study examined changes in biomarkers of exposure (BoE) after 5 days of nicotine-salt pod system (NSPS) use, compared with continuation of usual-cigarette smoking and cigarette abstinence, among adult combustible cigarette smokers.

**Aims and Methods:**

A randomized, open-label, parallel-cohort, confinement study of healthy adult smokers, naive to NSPS use, was conducted. Participants (*N* = 90) were randomized to six cohorts (*n* = 15 each): exclusive ad libitum use of NSPS (four flavors: Virginia Tobacco, Mint, Mango, Creme), continuation of usual-brand cigarette smoking, or cigarette abstinence. Total nicotine equivalents and BoE (NNN, NNAL, 3-HPMA, MHBMA, S-PMA, HMPMA, CEMA, 1-OHP, and COHb) were measured.

**Results:**

Eight non-nicotine BoEs, measured in urine, were reduced by an aggregate of 85.0% in the pooled NSPS cohort; increased by 14.4% in the cigarette cohort (*p* < .001 for pooled NSPS vs. cigarette); and reduced by 85.3% in the abstinence cohort (*p* > .05; 99.6% relative reduction between pooled NSPS vs. abstinence). Similar changes in individual BoEs were also observed (*p* < .001 for each BoE between pooled NSPS vs. cigarettes; and abstinence vs. pooled NSPS; *p* > .05 for each BoE between pooled NSPS vs. abstinence). Blood COHb decreased by 71.8% in the pooled NSPS cohort and 69.1% in the abstinence cohort (*p* > .05) and increased by 13.3% in the cigarette cohort (*p* < .001). Mean total urine nicotine equivalents increased in the pooled NSPS and cigarette cohorts by 9% and 26%, respectively, and did not significantly differ (*p* > .05).

**Conclusion:**

Complete switching from cigarettes to NSPS produced significant reductions in key non-nicotine BoEs associated with cigarette smoking.

**Implications:**

The results of this study concorded with evidence that complete switching from combustible cigarettes to tobacco and nontobacco-flavored vapor products may reduce exposure to key carcinogens and other toxicants known to be associated with tobacco-related diseases. Future research is needed to assess the long-term health effects of NSPS use. These results should not be interpreted to mean that the use of NSPS is without any risk, particularly for nonusers of tobacco products.

## Introduction

Combustible cigarette smoking remains the leading cause of preventable disease and death worldwide. Smokers are exposed to significant levels of carcinogens and other toxicants known to cause cancer and serious cardiovascular, respiratory, and reproductive harm.^1,2^ Such toxicants—or their surrogates in the form of analogs, degradants, and metabolites—are readily detectable in the urine and bloodstream of tobacco smokers and are collectively known as biomarkers of exposure (BoEs).^3,4^

A 2018 National Academies of Science, Engineering and Medicine consensus report and other reports have concluded that “there is substantial evidence that except for nicotine, under typical conditions of use, exposure to potentially toxic substances from e-cigarettes is significantly lower compared with combustible tobacco cigarettes.” ^5,6^ Evidence suggests that adult smokers who completely switch from combustible cigarettes to electronic nicotine delivery system (ENDS) products may also reduce short-term adverse health outcomes.^7,8^ Accordingly, complete switching from combustible cigarettes to ENDS has been endorsed by some US and international health organizations as a route for potential harm reduction for adult smokers who are unable or unwilling to quit tobacco use.^9^

ENDS compose a heterogeneous set of products with numerous devices of various operating designs currently available to consumers, and their potential role in tobacco harm reduction remains controversial due to gaps in evidence.^10–15^ To better understand the risk profile of individual ENDS products, it is critical to characterize how each design affects user exposure to potential toxicants. The JUUL nicotine-salt pod system (NSPS; JUUL Labs, Inc., San Francisco, CA) is a fully closed ENDS that delivers aerosol to the user through the vaporization of an e-liquid containing propylene glycol, glycerol, flavorants, nicotine, and benzoic acid. A temperature control system integrated into the breath-actuated inhalation pathway is designed to maintain a consistent operating temperature, independent of puff intensity, to minimize the creation of combustion-related by-products under a range of conditions, including “dry wick.” ^16^ Thus far, data assessing exposure to key BoEs among adult smokers who switch to NSPS has been limited to demonstrations of reduced exposure to carbon monoxide associated with acute use of NSPS versus combusted cigarettes, and indirectly through toxicological studies comparing harmful and potentially harmful constituent (HPHC) emissions of NSPS versus combusted cigarettes.^17–19^

This randomized, open-label, parallel-cohort study was designed to evaluate whether exposure to key toxicants would be reduced when adult smokers completely switch from their usual cigarette brand to exclusive use of the NSPS during confinement over 5 days in a controlled environment. As a secondary aim, the study compared changes in 5-day values for each BoE between the cohorts of smokers assigned to NSPS, usual brand of combustible cigarette and smoking abstinence. We hypothesized that smokers who replaced their usual-brand combustible cigarettes with exclusive use of NSPS for 5 days (vs. continuing to smoke usual-brand combustible cigarette) would experience a significant reduction in non-nicotine BoEs. Post hoc analyses evaluating the reductions in non-nicotine BoEs observed between the NSPS and smoking abstinence cohorts were also conducted.

## Methods

### Participants

The study population (*N* = 90) consisted of healthy adult smokers aged 22–62 years who reported smoking 10 or more manufactured (king-size or 100s) combustible cigarettes per day for at least 12 months, were naive to use of ENDS products, and were willing to be confined to a clinical research setting (Celerion, Lincoln, NE) for the study duration. Participants were recruited from the area surrounding study site in Lincoln, NE. Smoking status was confirmed via urine cotinine (≥500 ng/mL) and exhaled carbon monoxide (>12 ppm) testing. Participants were excluded for presence of clinically significant health issues or illnesses that in the opinion of the Investigator would jeopardize the health of the subject or impact the validity of the study results (particularly, diabetes, asthma, chronic obstructive pulmonary disease, and cancer), body mass index > 40 kg/m^2^ or < 18 kg/m^2^, history of substance abuse, pregnancy or lactation, use of medications known to interact with cytochrome P450 (CYP) 2A6 within 14 days or 5 half-lives of the drug prior to check-in, use of nicotine products other than manufactured combusted cigarettes within 14 days prior to check-in, use of prescription smoking cessation treatments within 3 months prior to check-in, and donation of blood or blood components within 56 days prior to check-in.^20^ Participants were also excluded if they indicated unwillingness to use the NSPS products during the study after the initial product trial period. The study was carried out in accordance with Food and Drug Administration (FDA) and International Council on Harmonisation (ICH) guidelines regarding Good Clinical Practice and the ethical principles set forth in the Declaration of Helsinki. The study protocol and informed consent form were reviewed and approved by the Chesapeake Institutional Review Board (Columbia, MD). All participants provided informed consent and were compensated for their participation. The study was registered at https://clinicaltrials.gov (Identifier: NCT03463837).

### Study Design

**Figure 1.**
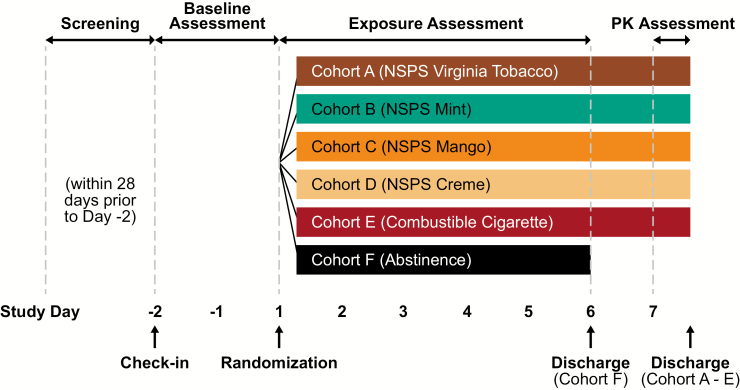
Overall study design.

Figure 1 depicts the study design and timeline. Eligible participants completed a randomized, open-label, parallel-cohort study while confined to an inpatient clinic over the course of 9 days. Participants were randomized into one of six product cohorts (n = 15 per cohort): (1) NSPS Virginia Tobacco flavor; (2) NSPS Mint flavor; (3) NSPS Mango flavor; (4) NSPS Creme flavor; (5) usual-brand combustible cigarette; and (6) smoking abstinence. Baseline assessments were made during a 2-day period while participants smoked their usual cigarettes. Beginning on the first day of the exposure assessment period, participants exclusively used their randomly assigned study product (i.e., NSPS or combustible cigarette) ad libitum in designated, supervised smoking areas, or underwent nicotine/tobacco product abstinence for five consecutive days. Urine samples were collected during the baseline assessment period and on the last day of the exposure assessment period, and blood samples were collected on each study day for analysis of BoEs. Subjective measures of urge to smoke and nicotine dependence were also assessed at baseline and at the end of the study.

### Study Procedure

Participant screening took place during the 28 days prior to the beginning of the study. Screening evaluations included a physical examination (vital signs and ECG), spirometry, urine alcohol and drug screening, exhaled carbon monoxide, urine cotinine assessment (CO > 12 ppm and urine cotinine ≥ 500 ng/mL), and serum pregnancy tests for females. At this time participants also completed questionnaires assessing demographic and smoking characteristics and were required to complete a 30-minute product trial in which they self-administered each of the four flavors of NSPS selected for this study.

Participants who successfully completed the screening evaluation were invited to enroll into the study. Upon arriving at the clinical research facility for check-in, additional safety evaluations were performed. Study participants had their personal belongings thoroughly examined and were required to shower and receive clean articles of clothing. All participants were allowed to smoke their usual-brand cigarette during the screening process until 23:00 on the day preceding randomization. Participants randomized to the NSPS or combustible cigarette cohorts began exclusive use of their assigned study product on each day of the exposure assessment period. Product use was ad libitum upon request to the study staff from 07:30 until 23:00 with limited exceptions (i.e., during meals or study assessments). NSPS pods that were fully consumed or failed to work properly were replaced by study staff, and new devices were provided to participants for product use on each study day. To avoid potential secondhand exposure, smoking and NSPS use were restricted to separate sections of the clinic. Further participants randomized to the abstinence cohort did not use any tobacco/nicotine-containing products throughout the 5-day assessment period and were housed separately from the other smoking and NSPS cohorts.

Total voided urine samples were collected during 24-hour time periods per subject per day at baseline (from 07:30 on Day −1 until 07:30 on Day 1) and following 5 days of product use (from 07:30 on Day 5 until 07:30 on Day 6). All urine collected during each 24-hour interval were pooled together and weighed. Blood samples were collected via direct venipuncture daily from Day −1 through Day 5 at approximately 19:00 and were preceded by a minimum 15-minute abstention from study product use. NSPS consumption during each day was quantified by comparing pod weight before and after use. On Day −1 and Day 5, participants also completed measures of urge to smoke and nicotine dependence at approximately 20:00.

Nonabstinence cohorts participated in further evaluation of pharmacokinetics over two additional study days; results will be discussed in future publications.

### Test Products

The four JUUL NSPS products evaluated in this study were closed systems consisting of the rechargeable device and disposable pods pre-filled with 0.7 mL of 5% nicotine-salt solution by weight (0.77 g of e-liquid per pod, 59 mg/mL of nicotine, i.e., 40 mg nicotine per pod).^16^ NSPS products comprised four commercially available flavors: Virginia Tobacco, Mint, Mango, and Creme. Participants randomized to the usual-brand combustible cigarette cohort provided their usual-brand combustible cigarette in unopened packs at study check-in. Unused cigarettes were returned to each participant at study completion, and participants were reimbursed for the cost of cigarettes consumed during the study. All products were stored in a locked, limited-access area in the study site and kept at controlled room temperature (15–30°C [59–86°F]).

### BoE Evaluation and Quantification

BoE analytes collected in urine for primary outcome measures included *N*-nitrosonornicotine (NNN), 4-(methylnitrosamino)-1-(3-pyridyl)-1-butanol (NNAL), 3-hydroxypropyl mercapturic acid (3-HPMA), monohydroxybutenylmercapturic acid (MHBMA), and S-phenylmercapturic acid (S-PMA); carboxyhemoglobin (COHb) was collected in blood and hydroxymethyl propylmercapturic acid (HMPMA), 2-cyanoethylmercapturic acid (CEMA), and 1-hydroxypyrene (1-OHP) in urine. A list of BoEs and their parent compounds is provided in Supplementary Table S3. Nicotine equivalents (nicotine, cotinine, trans-3’hydroxycotinine, and glucuronides) were collected in urine, and nicotine, cotinine, and trans-3′hydroxycotine were measured in blood as secondary outcome measures. The panel of BoEs selected represents chemicals or classes of chemicals identified by FDA as HPHCs and has been reported previously.^8,21–24^

Each BoE was measured using validated methods by Celerion (Lincoln, NE) based on FDA’s Guidance to Industry for Bioanalytical Method Validation (2001), Good Laboratory Practices (per 21 CFR Part 58), and the EMEA Guideline on Bioanalytical method validation (EMEA/CHMP/EWP/192217/2009 Rev. 1 Corr.2). Urine BoEs were analyzed using HPLC-MSMS and COHb was analyzed using spectrophotometry.

The urine biomarker concentrations were converted into biomarker quantities excreted in 24 hours by multiplying the measured concentration by the total volume of urine produced by the subject in the 24-hour period. Absolute change and percent change from baseline were calculated by referencing baseline (total mass excreted over 24 hours recovered at baseline) and post-baseline (Days 5–6) measurements as follows:

$$\begin{array}{ll} Absolute\;change\;from\;baseline\;=\; & \left(Post\text{-}baseline\;total\;mass\right)\\ & -(baseline\;total\;mass) \end{array}$$

$$\begin{array}{ll} Percent\;change\;from\;baseline\;=\; & \left([absolute\;change\;from\;baseline\right]\\ & /(baseline\;total\;mass])\;\times \;100 \end{array}$$

Note that the absolute and percent change from baseline were calculated at the individual subject level prior to summary (i.e., mean, median, range, CI, etc.). However, when reporting pooled results across all BoEs, the statistics (i.e., mean) at the individual BoE level were further combined (i.e., averaged) to obtain an overall summary.

### Nicotine Dependence and Urge to Smoke

At the time of check-in of clinic confinement, participants completed the Fagerström Test of Cigarette Dependence, a six-item measure of cigarette-based nicotine dependence on a 10-point scale, for characterization of baseline nicotine dependence. The total cigarette dependence score from the brief version (37 items) of the Wisconsin Inventory of Smoking Dependence Motives (WISDM) was administered at 20:00 on Day −1 and Day 5 to assess potential changes in factors influencing dependence during the study.^25^ Severity of urge to smoke was assessed on a 100-point visual analog scale with anchors of “Not at all” and “Extremely” at approximately 20:00 on Day −1 through Day 5.

### Adverse Events

Incidence of adverse events (AEs), and other safety end points, including vital signs, clinical laboratory tests, and physical examinations, as well as NSPS device malfunction or misuse, were also evaluated over the study period. The severity of each AE was rated by the investigator using a 3-point scale of mild, moderate, or severe.

### Data Analysis

The study was powered to assess changes in primary urine and blood BoEs following a 5-day, exclusive-use period of four NSPS cohorts relative to baseline. Assuming the reduction trends in BoEs observed from prior publication,^4^ 10 subjects would be sufficient to detect a significant decrease in any primary BoE with a minimum of 96% power at the one-sided .05 alpha level (Supplementary Tables S1). Primary analyses utilized one-tailed paired *t*-tests to compare the change from baseline values for total urinary NNN, NNAL, 3-HPMA, MHBMA, and S-PMA mass excreted, blood COHb, and secondary BoEs across each NSPS cohort. In addition, a linear mixed model analysis of variance compared all between-cohort differences in the absolute change from baseline values of each of the primary and secondary BoEs. The within-cohort differences in urge to smoke values, and Brief-WISDM subscales, total score from baseline to Day 5 were compared using two-tailed paired *t*-tests. Data analyses were carried out using SAS software version 9.3 (SAS Institute, Cary, NC) with differences considered statistically significant at an alpha level of .05.

## Results

### Participant Accrual and Demographic Characteristics

Between February and July 2018, 180 volunteers were screened and 90 subjects (56 males and 34 females) were enrolled and randomized to the six study cohorts (n = 15 each). Approximately a quarter of ineligible participants (26.1%) failed laboratory screening, 17.0% failed vitals, 17.0% did not agree to study confinement restriction, 9.1% failed spirometry, 6.8% failed the exhaled carbon monoxide breath test, and the remaining 24.0% had a scheduling conflict or did not meet other study eligibility criteria. Demographics and baseline characteristics were similar between cohorts (Table 1). The sample was composed of adult smokers (*M* [*SD*] age = 39.1 [11.4]), who were predominantly male (62.0%) and Caucasian (80.0%). Subjects reported, on average, moderate nicotine dependence on the Fagerström Test of Cigarette Dependence (M [*SD*] = 5.3 [1.6]) and smoked 16.2 (*SD* = 3.6) cigarettes per day. All participants randomized to the use of NSPS (n = 60) and usual cigarettes (n = 15) completed the study. Four participants from the abstinence arm terminated early (n = 11; 73% completion rate in-arm) for personal reasons.

**Table 1.** Baseline demographics and characteristics (safety population)^a^

Parameters	NSPS cohorts				Pooled	Combustible cigarette	Abstinence	Overall
	VT	Mint	Mango	Creme				
*n*	15	15	15	15	60	15	15	90
Age, y	37.1 ± 7.8	35.5 ± 13.1	41.2 ± 10.0	42.1 ± 11.4	39.0 ± 10.9	40.3 ± 11.1	38.6 ± 14.3	39.1 ± 11.4
Sex, male %	10 (67)	10 (67)	9 (60)	8 (53)	37 (62)	9 (60)	10 (67)	56 (62)
Race								
American Indian or Alaska Native	0 (0)	0 (0)	0 (0)	0 (0)	0 (0)	1 (7)	1 (7)	2 (2)
Black or African American	2 (13)	1 (7)	3 (20)	3 (20)	9 (15)	1 (7)	3 (20)	13 (14)
White	12 (80)	14 (93)	11 (73)	12 (80)	49 (82)	13 (87)	10 (67)	72 (80)
Other/multiple	1 (7)	0 (0)	1 (7)	0 (0)	2 (4)	0 (0)	1 (7)	3 (3)
Ethnicity								
Hispanic or Latino	0 (0)	0 (0)	1 (7)	1 (7)	2 (3)	2 (13)	0 (0)	4 (4)
Not Hispanic or Latino	15 (100)	15 (100)	14 (93)	14 (93)	58 (97)	13 (87)	15 (100)	86 (96)
BMI, kg/m^2^	26.9 ± 5.8	27.1 ± 4.4	29.8 ± 5.6	29.1 ± 5.5	28.2 ± 5.3	27.8 ± 4.3	27.5 ± 5.7	28.0 ± 5.2
Cigarette dependence (FTCD)	5.4 ± 1.6	5.5 ± 1.4	5.3 ± 1.4	5.8 ± 1.8	5.5 ± 1.5	4.9 ± 1.7	5.2 ± 1.4	5.3 ± 1.6
Cigarettes per day	15.8 ± 3.0	15.4 ± 3.4	16.8 ± 3.5	17.9 ± 5.0	16.5 ± 3.8	15.1 ± 3.7	16.2 ± 2.6	16.2 ± 3.6

BMI = body mass index; VT = Virginia Tobacco; FTCD = Fagerström Test for Cigarette Dependence (range: 0–10); NSPS = nicotine-salt pod system.

^a^Data are presented as *n* (%) or mean ± *SD*.

### Nicotine Intake and Product Consumption

**Figure 2.**
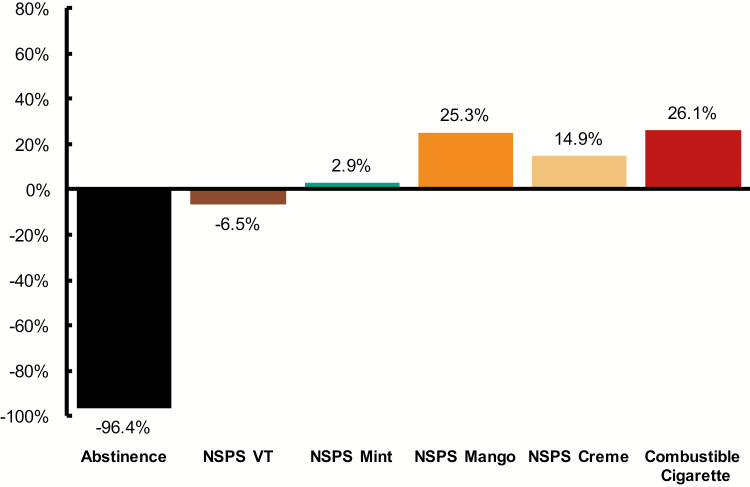
Mean change in total nicotine equivalents (Day 5 vs. Baseline). Data are presented as percent change: %, Day 5 vs. Baseline = ([Day 5 − Baseline]/Baseline) × 100. VT = Virginia Tobacco.

Across the 5-day study period, mean total urine nicotine equivalents (mg/24 hr) decreased by 96.4% in the abstinence cohort and increased in both the pooled NSPS (+9.2%, calculated at the population level) and usual-cigarette cohorts (+26.1%) (NS [*p* > .05] for pooled NSPS vs. usual cigarette; Figure 2). Change in mean total nicotine equivalents was closest to the usual-cigarette cohort in the Mango NSPS cohort (increase of 25.3%) and was statistically significant (*p* = .045). For Creme (+14.9%), Mint (+2.9%), and Virginia Tobacco (decrease of 6.5%), the change from baseline observed was not statistically significant.

On Days 4–5, the pooled NSPS cohorts consumed an average of 0.79 g per day of e-liquid (0.39 *SD*; Supplementary Table S2). On Day 5, mean total urine nicotine equivalents were 18.3 mg/24 hr (10.2 *SD*) for the pooled NSPS cohorts. The usual-brand combustible cigarette cohort consumed an average of 19.3 cigarettes per day (4.87 *SD*) and had total urine equivalents of 19.0 mg/24 hr (4.5 *SD*). Under these experimental conditions, consumption of one pod equivalent of e-liquid was associated with an observed concentration of total nicotine equivalents that was approximately 91% of the concentration associated with consumption of a pack of combustible cigarettes.

Estimated cigarette-pack total nicotine equivalents per pod consumed were similar across NSPS flavors, with Mint (85% cigarette-pack nicotine equivalents per pod) and Mango (89%) trending slightly lower than Virginia Tobacco (96%) and Creme (97%) cohorts.

### Change in Non-nicotine BoE

**Figure 3.**
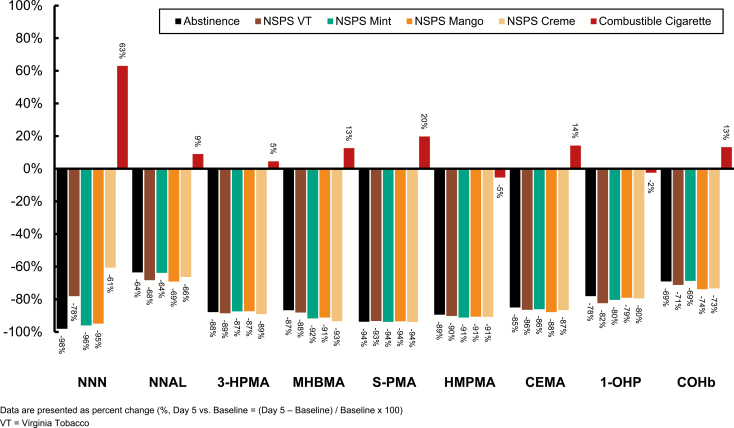
Percent change in biomarkers of exposure (mean percent change, Day 5 vs. Baseline).

All non-nicotine BoEs decreased relative to baseline in the abstinence and pooled NSPS cohorts. Conversely, all non-nicotine BoEs except 1-OHP and HMPMA increased in the usual-cigarette cohort (Figure 3; Supplementary Tables S4 and S5). Across cohorts and NSPS flavors, the mean percent reduction for each BoE was comparable between the pooled NSPS and abstinence cohorts (NS for each BoE). In aggregate, the eight urine BoEs were reduced by 85.3% in the abstinence cohort and 85.0% in the pooled NSPS cohort (NS), resulting in a 99.6% relative overall reduction in the BoEs studied (data were calculated at the population level). Comparatively, urine biomarkers in the cigarette cohort increased by an aggregate of 14.4% across all BoEs (pooled NSPS vs. usual cigarette; *p* < .001). Across nonsmoking cohorts, the reduction in NNAL was noticeably less than that of the other urine BoEs (Figure 3). This observation is consistent with the long terminal elimination half-life of NNAL (up to 45 days), thus maximal reduction after 5 days was not expected.^26^ In blood, COHb was reduced by 71.8% and 69.1% in the pooled NSPS and abstinence cohorts (NS), respectively; COHb increased by 13.3% in the cigarette cohort (pooled NSPS vs. usual cigarette; *p* < .001).

### Change in Urge to Smoke

As illustrated in Supplementary Figure S1 and Table S6, mean urge to smoke initially increased in the abstinence cohort, with maximal difference versus baseline of +28.9 units on Day 2. This increase diminished on subsequent days, with a Day 5 difference of +5.8 units (NS). In contrast, urge to smoke was consistently reduced relative to baseline in the usual-cigarette cohort, with maximal differences on Day 2 of −14.6 units and Day 5 of −11.5 (*p* = .04).

Mean differences in urge to smoke in the NSPS cohorts were intermediate between the usual-cigarette and abstinence cohorts, and trended closer to the usual-cigarette cohort on Day 2. Differences versus baseline in urge to smoke in the NSPS cohorts were not significant on Day 5.

### Change in WISDM Scale

Mean total WISDM scores at baseline ranged from 42.1 to 48.5 across all cohorts, indicating a moderate level of cigarette dependence (Supplementary Figure S2). Total dependence scores observed at Day 5 (vs. baseline) decreased in all NSPS cohorts (Difference = −3.46, 5.31) and in the abstinence cohort (Difference = −5.32; *p* = .04); however, there was a smaller decrease in the combustible cigarette cohort (Difference = −1.77). Mean scores in the primary dependence motives scale (automaticity, loss of control, craving, and tolerance) and secondary dependence motives scale (affiliative attachment, cognitive enhancement, cue exposure/associative processes, social/environmental goads, taste, weight control, and affective enhancement) were similar at baseline, ranging from 3.4 to 4.8 across cohorts. Scores were slightly decreased for all cohorts on Day 5, with the smallest decrease noted in the usual-cigarette cohort for both primary dependence motives and secondary dependence motives scales.

### Adverse and Serious Adverse Events

There were no serious AEs reported, and no subjects were discontinued due to AEs. There were 16 AEs reported by 14 of 90 subjects (16%) during baseline; the most frequently reported event was headache (*n* = 7; 8%). After randomization, 24 of 90 (27%) subjects across all study cohorts experienced a total of 36 AEs. One AE (irritability, in the smoking abstinence cohort) was moderate in severity and the remaining 35 AEs were mild. The most frequent was presyncope related to blood draws (8%) and all remaining events were experienced by three or fewer (≤3%) subjects overall (Supplementary Table S7).

## Discussion

This randomized controlled study found that after 5 days of substituting NSPS use for combustible cigarettes, the levels of nine clinically relevant non-nicotine BoEs (six primary and three secondary) significantly decreased and these reductions were comparable to those observed with smoking abstinence. The primary hypothesis of demonstrating a significant reduction in the six primary BoEs was successfully demonstrated in 23 of 24 (96%) comparisons at the individual NSPS cohort level (six BoEs and four NSPS cohorts) and in six of six (100%) comparisons at the pooled NSPS level. These results suggest that complete switching to NSPS from combustible cigarettes for 5 days is associated with reduction in tobacco-related BoEs and, by extension, reduced exposure to key carcinogens and other toxicants that present a health risk to smokers. These findings are congruent with results from previous studies of ENDS products that have found substantially reduced levels of BoE and carcinogens among combustible cigarette smokers who switched to ENDS.^4,27,28^

Over the course of the 5-day confinement period, the usual-brand combustible cigarette cohort experienced a 26% increase in total urine nicotine equivalents and, as expected, the abstinence cohort exhibited a marked decrease. During the study period, there was sustained nicotine intake across the pooled NSPS cohorts. Values in the Virginia Tobacco and Mint cohorts were lower than those observed in the Mango and Creme cohorts, consistent with the amount of product use based on the change in pod weight over the 5 days of the study. Among the NSPS cohorts, the Mango cohort most closely matched the increase in total nicotine equivalents seen in the usual-cigarette cohort, followed by Creme and Mint. This is consistent with data from previous studies demonstrating that flavored (vs. tobacco flavored) ENDS products are preferred by many adult smokers and may influence topography and consumption^29–32^; further research is necessary to examine the extent to which flavored NSPS products promote complete switching from cigarettes for adult smokers compared to tobacco flavors, as well as the net impact of flavored products on users and nonusers of tobacco products.

The toxic constituents of smoke from combusted tobacco have been widely described and well characterized.^14–16,33–37^ Other HPHCs are categorized as tobacco-specific nitrosamines, carbonyls, volatile organic compounds, polyaromatic hydrocarbons, and carbon monoxide. Select HPHCs have been identified by the FDA through published notice and guidance in accordance with Section 904 of the Food, Drug and Cosmetic Act. Although HPHCs are typically studied directly in aerosol chemistry, the metabolic analogues measured in human biomarker studies can inform the risk of specific types of diseases associated with HPHC exposure.^17^ Similarly, the aggregate changes in non-nicotine BoEs among NSPS and smoking abstinence cohorts observed within 5 days after completely abstaining from smoking are consistent with the design of the NSPS, which is temperature regulated to minimize generation of combustion-related degradation by-products. However, long-term studies directly assessing endpoints linked to smoking-related diseases are necessary to determine whether reductions in BoEs will translate into improvements in health.

Although median percent changes in NNN values were similar in the NSPS and abstention groups, unusually high NNN excretion values were noted on Day 5 for three participants in the NSPS and usual-cigarette cohorts, representing increases from baseline of approximately 168%, 426%, and 529%. In each case, the increase in NNN was not consistent with the changes observed for the other BoEs. Similar occasional increases have been reported previously in users of oral and dermal (i.e., patch) nicotine replacement therapies that have been attributed to endogenous nitrosation of nicotine and/or nornicotine in acidic environments (e.g., stomach), or in the presence of bacteria that catalyze nitrosation at neutral pH (e.g., oral cavity).^38–41^ The findings of these previous studies, coupled with the significant reductions observed in the other BoE for the subjects in question and lack of NNN increases in any other subjects switching to NSPS products, suggest that it is unlikely that NNN was delivered directly from the NSPS products.^18^ Further research is warranted to determine what specific factors, if any, may be associated with potential endogenous synthesis of NNN in consumers of ENDS and nicotine replacement therapy products.

Data from the self-reported measures of urge to smoke and nicotine dependence were mixed. The magnitude and direction of changes in urge to smoke varied across the NSPS cohorts; by Day 5 none of the comparisons differed significantly from baseline. Urge to smoke increased in the abstinence group and decreased in the usual-cigarette group; measures for the NSPS cohorts were intermediate but trended more closely to the usual-cigarette cohort, a similarity that may lend to a potential to substitute for usual cigarettes. The urge to smoke observations among the NSPS cohorts are generally consistent with a previous study, which also showed responses varying by ENDS flavor during a similar short-term switch, and the mixed nature of the responses may be due to randomization of the subjects to nonpreferred flavors, which may impact satisfaction.^4^

As measured by the Brief-WISDM questionnaire, smoking dependence decreased from Day 1 to Day 5, with several of the reductions reaching statistical significance, including the primary and secondary dependence motive scores and the total scores. As these changes were typically small in magnitude, and similar directional changes were also often observed in the smoking and abstinence cohorts, it is unclear whether these changes were the result of actual changes in factors motivating dependence, acute changes in withdrawal symptoms, or the conditions of confinement. However, 5 days may be insufficient to experience changes in nicotine dependence. Lower Brief-WISDM scores were also observed in smokers who were randomized to a 3-week switch to ENDS products compared with subjects who continued to smoke, both at the end of the 3-week switch and at the end of the 3-month follow-up period.^42^

The results of the study should be interpreted in light of several limitations. First, by design, this study was a short-term, highly controlled (100%) switch from combustible cigarettes to the NSPS or abstinence. Participants and their access to their respective test products were maintained in a controlled environment that may not reflect real-world consumption patterns or participant preferences, as participants were randomized to a particular NSPS flavor cohort and did not choose a flavor based on experimentation or personal preference. Furthermore, the convenience of ad libitum access to products in a clinic setting with fairly limited activities available may have given subjects the opportunity to consume more than they would have in a natural setting where cigarette smoking and vaping are banned in many places. Indeed, subjects randomized to continue smoking self-reported smoking 16.2 cigarettes per day at screening, 18.1 cigarettes per day during the baseline period, and 19.3 cigarettes per day on Days 4–5. That said, the structured environment provided quantitative results in a durable baseline format that may inform future studies on real-world use in which participants have the opportunity to choose their preferred flavor and have access to other tobacco- and nicotine-containing products. Future studies can address impact if any of, race, or other demographic factors on switching from cigarettes to NSPS.

Second, cross-cohort comparisons for BoE were not powered in the study design, and the potential effects of dual use were not examined. Long-term studies that include dual users may elucidate changes in other BoEs, biomarkers of potential harm, or precursors to disease processes that may result from prolonged reduction in exposure to toxicants found in cigarette smoke and determine the effects of dual use on exposure to nicotine and other toxicants.

Last, this study was not designed to evaluate whether there is an increase in non-nicotine BoE exposure in non-tobacco users who initiate NSPS use. This study should not be considered as suggesting that NSPS use is “safe” for non-tobacco of tobacco products.

In conclusion, this study has clearly demonstrated that complete obligatory switching from combustible cigarettes to NSPS for 5 days among a sample of adult smokers resulted in significant reductions in key tobacco-related BoEs, without significant changes in nicotine exposure parameters. These findings suggest that complete switching to NSPS may reduce exposure to key carcinogenic and toxic substances and could serve as a route for potential harm reduction for adult smokers who are unwilling or unable to quit tobacco use. Future research is needed to assess the real-world clinical relevance of changes in these BoEs and toxicant reductions to longer-term disease risk for adult smokers who switch to NSPS.

## Supplementary Material

ntz206_suppl_Suplemental_Table_S1

ntz206_suppl_Suplemental_Table_S2

ntz206_suppl_Suplemental_Table_S3

ntz206_suppl_Suplemental_Table_S4

ntz206_suppl_Suplemental_Table_S5

ntz206_suppl_Suplemental_Table_S6

ntz206_suppl_Suplemental_Table_S7

ntz206_suppl_Suplemental_Figure_1

ntz206_suppl_Suplemental_Figure_2

## References

[1] U.S. Department of Health and Human Services. The Health Consequences of Smoking – 50 Years of Progress: A Report of the Surgeon General. Atlanta, GA: U.S. Department of Health and Human Services, Centers for Disease Control and Prevention, National Center for Chronic Disease Prevention and Health Promotion, Office on Smoking and Health Published 2014 Accessed June 16, 2019.

[2] Wald NJ, Hackshaw AK Cigarette smoking: an epidemiological overview. Br Med Bull. 1996;52(1):3–11. 8746292 10.1093/oxfordjournals.bmb.a011530

[3] Mattes W, Yang X, Orr MS, Richter P, Mendrick DL Biomarkers of tobacco smoke exposure. Adv Clin Chem. 2014;67:1–45. 25735858 10.1016/bs.acc.2014.09.001

[4] D’Ruiz CD, Graff DW, Robinson E Reductions in biomarkers of exposure, impacts on smoking urge and assessment of product use and tolerability in adult smokers following partial or complete substitution of cigarettes with electronic cigarettes. BMC Public Health. 2016;16(1):543. 27401980 10.1186/s12889-016-3236-1 PMC4940751

[5] The National Academies of Science Engineering Medicine. Public Health Consequences of E-cigarettes. NASEM Consensus Report 2018. Published January 2018. http://nationalacademies.org/hmd/reports/2018/public-health-consequences-of-e-cigarettes.aspx Accessed June 16, 2019.

[6] McNeill A, Brose LS, Calder R, Hitchman SC, Hajek P, McRobbie H. E-cigarettes: An Evidence Update. A report commissioned by Public Health England. Published August. London: Public Health England; 2015. 10.1016/S0140-6736(15)00079-3 26338257

[7] Polosa R, Morjaria J, Caponnetto P, et al. Effect of smoking abstinence and reduction in asthmatic smokers switching to electronic cigarettes: evidence for harm reversal. Int J Environ Res Public Health. 2014;11(5):4965–4977. 24814944 10.3390/ijerph110504965 PMC4053879

[8] Goniewicz ML, Gawron M, Smith DM, Peng M, Jacob P 3rd, Benowitz NL Exposure to nicotine and selected toxicants in cigarette smokers who switched to electronic cigarettes: a longitudinal within-subjects observational study. Nicotine Tob Res. 2017;19(2):160–167. 27613896 10.1093/ntr/ntw160 PMC5234360

[9] Saitta D, Chowdhury A, Ferro GA, Nalis FG, Polosa R A risk assessment matrix for public health principles: the case for e-cigarettes. Int J Environ Res Public Health. 2017;14(4). doi:10.3390/ijerph14040363 10.3390/ijerph14040363 PMC5409564 28362360

[10] Goniewicz ML, Smith DM, Edwards KC, et al. Comparison of nicotine and toxicant exposure in users of electronic cigarettes and combustible cigarettes. JAMA Netw Open. 2018;1(8):e185937. 30646298 10.1001/jamanetworkopen.2018.5937 PMC6324349

[11] Lindson-Hawley N, Hartmann-Boyce J, Fanshawe TR, Begh R, Farley A, Lancaster T Interventions to reduce harm from continued tobacco use. Ireland, Europe: John Wiley & Sons Ltd; 2016. 10.1002/14651858.CD005231.pub3 PMC6463938 27734465

[12] Franck C, Filion KB, Kimmelman J, Grad R, Eisenberg MJ Ethical considerations of e-cigarette use for tobacco harm reduction. Respir Res. 2016;17(1):53. 27184265 10.1186/s12931-016-0370-3 PMC4869264

[13] Latif E, Nair M E-cigarettes: a need to broaden the debate. Int J Tuberc Lung Dis. 2016;20(11):1430–1435. 27776582 10.5588/ijtld.16.0524

[14] Fagerström KO, Bridgman K Tobacco harm reduction: the need for new products that can compete with cigarettes. Addict Behav. 2014;39(3):507–511. 24290207 10.1016/j.addbeh.2013.11.002

[15] Nollen NL, Mayo MS, Clark L, et al. Tobacco toxicant exposure in cigarette smokers who use or do not use other tobacco products. Drug Alcohol Depend. 2017;179(1):330–336. 28843083 10.1016/j.drugalcdep.2017.07.021 PMC5599364

[16] Gilman G, Johnson MS, Martin A, et al. Characterization of temperature regulation and HPHC profile of a nicotine salt-based ENDS product. In: SNRT Conference; 2018 JLIscience.com. Accessed June 2019.

[17] Gilman G, Johnson M, Martin A, et al. Characterization of temperature regulation and HPHC profile of a nicotine-salt based ENDS product. Poster session presented at: Society for Research on Nicotine and Tobacco Conference; February 21–24, 2018; Baltimore, MD.

[18] Gilman G, Johnson M, Martin A, et al. HPHC analysis of eight flavors of a temperature-regulated nicotine salt-based ENDS product. Poster session presented at: Global Forum on Nicotine Conference; 2018 June 14–16; Warsaw, Poland.

[19] Wynne C, Waaka DS, Devonie S.Acute use of nicotine salt-based ENDS and combusted cigarettes. Poster session presented at: Society for Research on Nicotine and Tobacco 24th Annual Meeting; 2018 February 21–24; Baltimore, MD.

[20] Lexicomp. Drug Information Handbook: A Clinically Relevant Resource for All Healthcare. 24th ed. Hudson, OH: Wolters Kluwer, Inc.; 2015.

[21] Leslie Kux. Harmful and Potentially Harmful Constituents in Tobacco Products and Tobacco Smoke; Established List. Food and Drug Administration https://www.fda.gov/tobacco-products/rules-regulations-and-guidance/harmful-and-potentially-harmful-constituents-tobacco-products-and-tobacco-smoke-established-list. Published April 2012. Accessed September 2019.

[22] Yan XS, D’Ruiz C Effects of using electronic cigarettes on nicotine delivery and cardiovascular function in comparison with regular cigarettes. Regul Toxicol Pharmacol. 2015;71(1):24–34. 25460033 10.1016/j.yrtph.2014.11.004

[23] Round EK, Chen P, Taylor AK, Schmidt E Biomarkers of tobacco exposure decrease after smokers switch to an e-cigarette or nicotine gum. Nicotine Tob Res. 2019;21(9):1239–1247. 30202883 10.1093/ntr/nty140 PMC6698949

[24] Hecht SS, Carmella SG, Kotandeniya D, et al. Evaluation of toxicant and carcinogen metabolites in the urine of e-cigarette users versus cigarette smokers. Nicotine Tob Res. 2015;17(6):704–709. 25335945 10.1093/ntr/ntu218 PMC4481723

[25] Smith SS, Piper ME, Bolt DM, et al. Development of the brief Wisconsin inventory of smoking dependence motives. Nicotine Tob Res. 2010;12(5):489–499. 20231242 10.1093/ntr/ntq032 PMC2861888

[26] Hecht SS, Carmella SG, Chen M, et al. Quantitation of urinary metabolites of a tobacco-specific lung carcinogen after smoking cessation. Cancer Res. 1999;59(3):590–596. 9973205

[27] Lorkiewicz P, Riggs DW, Keith RJ, et al. Comparison of urinary biomarkers of exposure in humans using electronic cigarettes, combustible cigarettes, and smokeless tobacco. Nicotine Tob Res. 2019;21(9):1228–1238. doi:10.1093/ntr/nty089 29868926 10.1093/ntr/nty089 PMC6698950

[28] Campbell LR, Brown BG, Jones BA, Marano KM, Borgerding MF Study of cardiovascular disease biomarkers among tobacco consumers, part 1: biomarkers of exposure. Inhal Toxicol. 2015;27(3):149–156. 25787703 10.3109/08958378.2015.1013228 PMC4496812

[29] St Helen G, Shahid M, Chu S, Benowitz NL Impact of e-liquid flavors on e-cigarette vaping behavior. Drug Alcohol Depend. 2018;189(1):42–48. 29879680 10.1016/j.drugalcdep.2018.04.032 PMC6211798

[30] St Helen G, Dempsey DA, Havel CM, Jacob P 3rd, Benowitz NL Impact of e-liquid flavors on nicotine intake and pharmacology of e-cigarettes. Drug Alcohol Depend. 2017;178(1):391–398. 28704768 10.1016/j.drugalcdep.2017.05.042 PMC5565733

[31] Robinson RJ, Hensel EC, Al-Olayan AA, Nonnemaker JM, Lee YO Effect of e-liquid flavor on electronic cigarette topography and consumption behavior in a 2-week natural environment switching study. PLoS One. 2018;13(5):e0196640. 29718974 10.1371/journal.pone.0196640 PMC5931659

[32] Koszowski B, Rosenberry ZR, Kanu A, Viray LC, Potts JL, Pickworth WB Nicotine and carbon monoxide exposure from inhalation of cigarillo smoke. Pharmacol Biochem Behav. 2015;139(Pt A):7–14. 26459155 10.1016/j.pbb.2015.10.007 PMC4662635

[33] Choi K, Sabado M, El-Toukhy S, Vogtmann E, Freedman ND, Hatsukami D Tobacco product use patterns, and nicotine and tobacco-specific nitrosamine exposure: NHANES 1999–2012. Cancer Epidemiol Biomarkers Prev. 2017;26(10):1525–1530. 28710077 10.1158/1055-9965.EPI-17-0338 PMC5626596

[34] Chang CM, Edwards SH, Arab A, Del Valle-Pinero AY, Yang L, Hatsukami DK Biomarkers of tobacco exposure: summary of an FDA-sponsored public workshop. Cancer Epidemiol Biomarkers Prev. 2017;26(3):291–302. 28151705 10.1158/1055-9965.EPI-16-0675 PMC5336443

[35] Conklin DJ, Malovichko MV, Zeller I, et al. Biomarkers of chronic acrolein inhalation exposure in mice: implications for tobacco product-induced toxicity. Toxicol Sci. 2017;158(2):263–274. 28482051 10.1093/toxsci/kfx095 PMC5837482

[36] Hecht SS, Stepanov I, Carmella SG Exposure and metabolic activation biomarkers of carcinogenic tobacco-specific nitrosamines. Acc Chem Res. 2016;49(1):106–114. 26678241 10.1021/acs.accounts.5b00472 PMC5154679

[37] Rostron BL, Chang CM, van Bemmel DM, Xia Y, Blount BC Nicotine and toxicant exposure among U.S. smokeless tobacco users: results from 1999 to 2012 National Health and Nutrition Examination Survey Data. Cancer Epidemiol Biomarkers Prev. 2015;24(12):1829–1837. 26582044 10.1158/1055-9965.EPI-15-0376 PMC5134927

[38] Jensen RP, Luo W, Pankow JF, Strongin RM, Peyton DH Hidden formaldehyde in e-cigarette aerosols. N Engl J Med. 2015;372(4):392–394. 25607446 10.1056/NEJMc1413069

[39] Hajek P, Phillips-Waller A, Przulj D, et al. A randomized trial of e-cigarettes versus nicotine-replacement therapy. N Engl J Med. 2019;380(7):629–637. 30699054 10.1056/NEJMoa1808779

[40] Stepanov I, Carmella SG, Briggs A, et al. Presence of the carcinogen N′-nitrosonornicotine in the urine of some users of oral nicotine replacement therapy products. Cancer Res. 2009;69(21):8236–8240. 19843845 10.1158/0008-5472.CAN-09-1084 PMC2783463

[41] Stepanov I, Carmella SG, Han S, et al. Evidence for endogenous formation of N′-nitrosonornicotine in some long-term nicotine patch users. Nicotine Tob Res. 2009;11(1):99–105. 19246447 10.1093/ntr/ntn004 PMC2734288

[42] Smith TT, Wahlquist AE, Heckman BW, Cummings KM, Carpenter MJ Impact of e-cigarette sampling on cigarette dependence and reinforcement value. Nicotine Tob Res. 2018. doi:10.1093/ntr/nty258 10.1093/ntr/nty258 PMC7297108 30500925

