# Supplementary material for: Five-Day Changes in Biomarkers of Exposure Among Adult Smokers After Completely Switching From Combustible Cigarettes to a Nicotine-Salt Pod System
Source: Nicotine Tob Res. 2019 Nov 5;22(8):1285–93. doi: 10.1093/ntr/ntz206 (PMC7364828; doi:10.1093/ntr/ntz206)
Supplement: ntz206_suppl_Suplemental_Table_S1 [file ntz206_suppl_suplemental_table_s1.docx]

Table S1**.** Estimated Power Requirements to Detect a Difference in Primary BOEs

| Biomarker | Units | Parameter Estimates^*^ | Power (n=10) | Power (n = 15) |
| --- | --- | --- | --- | --- |
| Urine NNN | (ng/24h) | -14.1 ± 11.9 | 96.4% | 99.7% |
| Urine NNAL | (ng/24h) | -227.0 ± 114.0 | >99.9% | >99.9% |
| Urine 3-HPMA | (ng/24h) | -1328.0 ± 834.0 | 99.8% | >99.9% |
| Urine MHBMA | (ng/24h) | -4.8 ± 3.4 | 99.3% | >99.9% |
| Urine S-PMA | (ng/24h) | -6.5 ± 4.0 | 99.7% | >99.9% |
| Blood COHb | (%) | -4.9 ± 1.8 | >99.9% | >99.9% |

*Parameter estimates based on D’Ruiz et. al., 2016
