# Supplementary material for: Five-Day Changes in Biomarkers of Exposure Among Adult Smokers After Completely Switching From Combustible Cigarettes to a Nicotine-Salt Pod System
Source: Nicotine Tob Res. 2019 Nov 5;22(8):1285–93. doi: 10.1093/ntr/ntz206 (PMC7364828; doi:10.1093/ntr/ntz206)
Supplement: ntz206_suppl_Suplemental_Table_S2 [file ntz206_suppl_suplemental_table_s2.docx]

Table S2: Relative Mean Product Consumption and Mean Total Nicotine Equivalents Excreted

| Measure | Units | NSPS Cohorts | | | | Pooled | Combustible Cigarette |
| --- | --- | --- | --- | --- | --- | --- | --- |
|  |  | VT | Mint | Mango | Creme |  |  |
| Number of participants | ITT participants | n=15 | n=15 | n=15 | n=15 | n=60 | n=15 |
| Mean e-liquid consumed / day (days 4-5) | Grams (actual, mean ± SD) | 0.657 ± 0.238 | 0.720 ± 0.238 | 0.964 ± 0.417 | 0.824 ± 0.530 | 0.79 ± 0.39 |  |
| Mean cigarettes consumed / day  (days 4-5) | Num. cigarettes (actual) |  |  |  |  |  | 19.3 ± 4.87 |
| Mean pod equivalents consumed / day | Pod content equivalents (estimated; assuming 0.77 g per pod^*^) | 0.85 ± 0.31 | 0.94 ± 0.31 | 1.25 ± 0.54 | 1.07 ± 0.69 | 1.03 ± 0.50 |  |
| Mean pack equivalents consumed / day | Pack equivalents  (actual; assuming 20 cigarettes per pack) |  |  |  |  |  | 0.97 ± 0.23 |
| Mean total nicotine equivalents excreted (day 5) | mg / 24h (actual) | 15.9 ± 7.8 | 15.5 ± 7.7 | 21.6 ± 9.8 | 20.2 ± 13.8 | 18.3 ± 10.2 | 19.0 ± 4.5 |
| Relative total nicotine equivalents excreted per pod or cigarette pack consumed | ^**^(usual cigarette = 100%) | ~96%^*^ | ~85%^*^ | ~89%^*^ | ~97%^*^ | ~91%^*^ | 100%^**^ |

^*^assumed e-liquid contents of pod are 100% consumed

VT = Virginia Tobacco
