# Supplementary material for: Five-Day Changes in Biomarkers of Exposure Among Adult Smokers After Completely Switching From Combustible Cigarettes to a Nicotine-Salt Pod System
Source: Nicotine Tob Res. 2019 Nov 5;22(8):1285–93. doi: 10.1093/ntr/ntz206 (PMC7364828; doi:10.1093/ntr/ntz206)
Supplement: ntz206_suppl_Suplemental_Table_S3 [file ntz206_suppl_suplemental_table_s3.docx]

Table S3: Summary of Biomarkers and Associated HPHCs Measured in this Study

| Biomarker | Chemical Name | Associated HPHC | HPHC Category | Risk Association | | | |
| --- | --- | --- | --- | --- | --- | --- | --- |
|  |  |  |  | CA^*^ | CT | RT | RDT |
| NNN | N-nitrosonornicotine | NNN | TSNA | **x** |  |  |  |
| NNAL | 4-(methylnitrosamino)-1-(3-pyridyl)-1-butanol | NNK | TSNA | **x** |  |  |  |
| 3-HPMA | 3-hydroxypropyl-mercapturic acid | acrolein | carbonyl |  | **x** | **x** |  |
| MHBMA | monohydroxylbutenyl-mercapturic acid | 1,3-butadiene | VOC | **x** |  | **x** | **x** |
| S-PMA | S-Phenyl-mercapturic acid | benzene | VOC | **x** | **x** |  | **x** |
| HMPMA | 3-Hydroxy-1-methylpropyl-mercapturic acid | crotonaldehyde | carbonyl | **x** |  |  |  |
| CEMA | 2-Cyanoethylmercapturic acid | acrylonitrile | VOC | **x** |  | **x** |  |
| 1-OHP^**^ | 1-hydroxypyrene | pyrene | PAH |  |  |  |  |
| COHb | Carboxyhemoglobin | carbon monoxide | carbonyl |  | **x** |  | **x** |

TSNA = Tobacco-specific nitrosamines, HPHC = harmful and potentially harmful constituents, NSK = Nicotine-derived nitrosamine ketone ^*^CA = carcinogen, CT = cardiovascular toxicant, RT = respiratory toxicant, RDT = reproductive and developmental toxicant

^**^Surrogate for exposure to polycyclic aromatic hydrocarbons.
