# Supplementary material for: Five-Day Changes in Biomarkers of Exposure Among Adult Smokers After Completely Switching From Combustible Cigarettes to a Nicotine-Salt Pod System
Source: Nicotine Tob Res. 2019 Nov 5;22(8):1285–93. doi: 10.1093/ntr/ntz206 (PMC7364828; doi:10.1093/ntr/ntz206)
Supplement: ntz206_suppl_Suplemental_Table_S4 [file ntz206_suppl_suplemental_table_s4.docx]

Table S4: Summary of Pairwise Change from Baseline (Day 5 – Baseline) in Blood and Urine Primary Biomarkers

| Parameters | NSPS Cohorts | | | | Pooled | Combustible Cigarettes | Abstinence |
| --- | --- | --- | --- | --- | --- | --- | --- |
|  | VT | Mint | Mango | Creme |  |  |  |
| n | 15 | 15 | 15 | 15 | 60 | 15 | 11 |
| Urine Biomarkers (Pairwise Change from Baseline: Day 5 - Baseline)^a^ | | | | |  | | |
| NNN | -11.9 (15.9)  [-20.7 to -3.1] | -12.8 (7.0)  [-16.7 to -9.0] | -12.4 (5.9)  [-15.7 to -9.1] | -1.7 (64.4)  [-37.4 to 34.0] | -9.7 (33.0)  [-18.2 to -1.2] | 14.3 (40.7)  [-8.3 to 36.8] | -20.0 (27.2)  [-38.3 to -1.7] |
| NNAL | -314.2 (155.5)  [-400.4 to -228.1] | -246.3 (139.0)  [-323.3 to -169.3] | -340.2 (155.8)  [-426.4 to -253.9] | -353.4 (116.7)  [-418.0 to -288.8] | -313.5 (145.1)  [-351.0 to -276.1] | 23.6 (111.2)  [-38.1 to 85.2] | -281.4 (203.9)  [-418.4 to -144.4] |
| 3-HPMA | -1.54 (0.58)  [-1.86 to -1.22] | -1.52 (0.60)  [-1.85 to -1.19] | -1.65 (0.60)  [-1.99 to -1.32] | -1.95 (0.97)  [-2.49 to -1.41] | -1.67 (0.71)  [-1.85 to -1.48] | 0.05 (0.30)  [-0.12 to 0.22] | -1.55 (0.73)  [-2.04 to -1.06] |
| MHBMA | -4.9 (4.5)  [-7.3 to -2.4] | -5.5 (3.9)  [-7.6 to -3.3] | -4.1 (2.5)  [-5.5 to -2.7] | -6.3 (5.6)  [-9.4 to -3.3] | -5.2 (4.2)  [-6.3 to -4.1] | 0.8 (1.5)  [-0.0 to 1.7] | -4.3 (4.0)  [-7.0 to -1.6] |
| S-PMA | -6.8 (5.3)  [-9.7 to -3.9] | -7.2 (4.8)  [-9.8 to -4.6] | -5.6 (2.5)  [-7.0 to -4.2] | -8.5 (7.9)  [-12.8 to -4.1] | -7.0 (5.4)  [-8.4 to -5.6] | 1.2 (1.7)  [0.3 to 2.2] | -5.6 (4.3)  [-8.5 to -2.8] |
| Blood Biomarkers (Pairwise Change from Baseline: Day 5 - Baseline)^a^ | | | | |  | | |
| COHb | -4.9 (1.9)  [-6.0 to -3.8] | -4.8 (1.7)  [-5.7 to -3.9] | -5.4 (1.5)  [-6.2 to -4.5] | -5.6 (2.3)  [-6.9 to -4.3] | -5.1 (1.9)  [-5.6 to -4.7] | 0.8 (1.4)  [+0.0 to 1.6] | -4.6 (1.7)  [-5.7 to -3.4] |

^a^ For each biomarker, data are presented as mean ± standard deviation and 95%-2-Sided-CI in the first and second rows, respectively. Note that the 2-Sided 95% CIs testing for no change (from baseline) are based on a paired T-test.

VT = Virginia Tobacco
