# Supplementary material for: Five-Day Changes in Biomarkers of Exposure Among Adult Smokers After Completely Switching From Combustible Cigarettes to a Nicotine-Salt Pod System
Source: Nicotine Tob Res. 2019 Nov 5;22(8):1285–93. doi: 10.1093/ntr/ntz206 (PMC7364828; doi:10.1093/ntr/ntz206)
Supplement: ntz206_suppl_Suplemental_Table_S5 [file ntz206_suppl_suplemental_table_s5.docx]

Table S5: Summary of Pairwise Change from Baseline (Day 5 – Baseline) in Blood and Urine Primary Biomarkers

| Parameters | | | NSPS Cohorts | | | | | Combustible Cigarette (N=15) | Abstinence  (N=11) |
| --- | --- | --- | --- | --- | --- | --- | --- | --- | --- |
|  |  |  | VT  (N=15) | Mint  (N=15) | Mango  (N=15) | Creme  (N=15) | Pooled  (N=60) |  |  |
| Urine NNN | (Units) | Summary |  | | | | | | |
| Day -1 (BL) | (ng over 24h) | Mean (SD) | 15.9 (10.2) | 13.3 (7.2) | 13.0 (6.0) | 20.8 (18.8) | 15.8 (11.8) | 16.5 (9.4) | 18.2 (23.7) |
| Day 5 | (ng over 24h) | Mean (SD) | 4.0 (13.5) | 0.5 (0.4) | 0.6 (0.3) | 19.1 (71.0) | 6.1 (36.1) | 30.8 (45.5) | 0.2 (0.2) |
| Difference (absolute)* | (ng over 24h) | Mean (SD) | -11.9 (15.9) | -12.8 (7.0) | -12.4 (5.9) | -1.7 (64.4) | -9.7 (33.0) | 14.3 (40.7) | -20.0 (27.2) |
|  | (ng over 24h) | Median | -10.1 | -12.4 | -13.7 | -12.2 | -12.3 | 2.6 | -9.6 |
|  | (ng over 24h) | 95% CI^***^ | [-20.7 to -3.1] | [-16.7 to -9.0] | [-15.7 to -9.1] | [-37.4 to 34.0] | [-18.2 to -1.2] | [-8.3 to 36.8] | [-38.3 to -1.7] |
| Difference (% reduction)^**^ | (% reduction) | Mean (SD) | -78.1 (68.3) | -96.0 (2.1) | -94.9 (3.2) | -60.6 (134.6) | -82.4 (75.0) | 63.0 (134.3) | -98.1 (1.6) |
|  | (% reduction) | Median | -96.8 | -96.6 | -96.2 | -96.1 | -96.3 | 26.4 | -98.5 |
|  | (% reduction) | 95% CI | [-115.9 to -40.3] | [-97.2 to -94.9] | [-96.6 to -93.1] | [-135.2 to 13.9] | [-101.8 to -63.0] | [-11.4 to 137.4] | [-99.2 to -97.0] |
| Urine NNAL | (Units) | Summary |  | | | | | | |
| Day -1 (BL) | (ng over 24h) | Mean (SD) | 458.8 (227.0) | 377.9 (195.0) | 490.9 (215.6) | 529.7 (177.5) | 464.3 (207.2) | 553.9 (229.6) | 481.1 (293.2) |
| Day 5 | (ng over 24h) | Mean (SD) | 144.5 (79.9) | 131.6 (71.0) | 150.7 (79.8) | 176.3 (76.4) | 150.8 (76.7) | 577.4 (201.7) | 159.5 (117.4) |
| Difference (absolute) | (ng over 24h) | Mean (SD) | -314.2 (155.5) | -246.3 (139.0) | -340.2 (155.8) | -353.4 (116.7) | -313.5 (145.1) | 23.6 (111.2) | -281.4 (203.9) |
|  | (ng over 24h) | Median | -285.4 | -249.8 | -300.4 | -363.4 | -296.3 | 37.1 | -241.4 |
|  | (ng over 24h) | 95% CI | [-400.4 to -228.1] | [-323.3 to -169.3] | [-426.4 to -253.9] | [-418.0 to -288.8] | [-351.0 to -276.1] | [-38.1 to 85.2] | [-418.4 to -144.4] |
| Difference (% reduction) | (%) | Mean (SD) | -68.3 (8.9) | -63.8 (15.1) | -69.2 (8.4) | -66.4 (9.3) | -66.9 (10.7) | 9.0 (19.2) | -63.5 (11.7) |
|  | (%) | Median | -70.8 | -66.0 | -68.3 | -69.3 | -68.4 | 7.4 | -66.5 |
|  | (%) | 95% CI | [-73.2 to -63.3] | [-72.2 to -55.4] | [-73.9 to -64.6] | [-71.5 to -61.3] | [-69.7 to -64.2] | [-1.6 to 19.6] | [-71.4 to -55.7] |
| Urine 3-HPMA | (Units) | Summary |  | | | | | | |
| Day -1 (BL) | (mg over 24h) | Mean (SD) | 1.73 (0.59) | 1.71 (0.61) | 1.87 (0.62) | 2.15 (0.98) | 1.87 (0.72) | 1.73 (0.47) | 1.80 (0.81) |
| Day 5 | (mg over 24h) | Mean (SD) | 0.19 (0.06) | 0.19 (0.05) | 0.22 (0.06) | 0.20 (0.05) | 0.20 (0.06) | 1.78 (0.47) | 0.19 (0.06) |
| Difference (absolute) | (mg over 24h) | Mean (SD) | -1.54 (0.58) | -1.52 (0.60) | -1.65 (0.60) | -1.95 (0.97) | -1.67 (0.71) | 0.05 (0.30) | -1.55 (0.73) |
|  | (mg over 24h) | Median | -1.47 | -1.31 | -1.50 | -1.77 | -1.58 | 0.12 | -1.57 |
|  | (mg over 24h) | 95% CI | [-1.86 to -1.22] | [-1.85 to -1.19] | [-1.99 to -1.32] | [-2.49 to -1.41] | [-1.85 to -1.48] | [-0.12 to 0.22] | [-2.04 to -1.06] |
| Difference (% reduction) | (%) | Mean (SD) | -88.6 (4.8) | -87.4 (4.7) | -87.3 (4.3) | -89.1 (5.2) | -88.1 (4.7) | 4.5 (17.2) | -87.9 (4.2) |
|  | (%) | Median | -89.6 | -87.0 | -87.1 | -91.7 | -88.7 | 7.5 | -88.1 |
|  | (%) | 95% CI | [-91.3 to -85.9] | [-90.0 to -84.8] | [-89.7 to -85.0] | [-92.0 to -86.2] | [-89.3 to -86.9] | [-5.0 to 14.0] | [-90.7 to -85.1] |
| Urine MHBMA | (Units) | Summary |  | | | | | | |
| Day -1 (BL) | (μg over 24h) | Mean (SD) | 5.0 (4.5) | 5.6 (3.9) | 4.3 (2.5) | 6.5 (5.6) | 5.4 (4.2) | 5.4 (3.2) | 4.6 (3.6) |
| Day 5 | (μg over 24h) | Mean (SD) | 0.2 (0.1) | 0.2 (0.1) | 0.2 (0.1) | 0.2 (0.1) | 0.2 (0.1) | 6.2 (4.1) | 0.2 (0.1) |
| Difference  (absolute) | (μg over 24h) | Mean (SD) | -4.9 (4.5) | -5.5 (3.9) | -4.1 (2.5) | -6.3 (5.6) | -5.2 (4.2) | 0.8 (1.5) | -4.3 (4.0) |
|  | (μg over 24h) | Median | -4.1 | -5.8 | -4.2 | -5.3 | -5.1 | 0.3 | -3.1 |
|  | (μg over 24h) | 95% CI | [-7.3 to -2.4] | [-7.6 to -3.3] | [-5.5 to -2.7] | [-9.4 to -3.3] | [-6.3 to -4.1] | [-0.0 to 1.7] | [-7.0 to -1.6] |
| Difference  (% reduction) | (% reduction) | Mean (SD) | -88.2 (16.4) | -91.7 (12.5) | -91.2 (11.4) | -93.4 (12.3) | -91.1 (13.1) | 12.6 (21.8) | -86.7 (18.1) |
|  | (% reduction) | Median | -96.3 | -96.5 | -95.4 | -97.6 | -96.3 | 8.8 | -93.9 |
|  | (% reduction) | 95% CI | [-97.2 to -79.1] | [-98.6 to -84.8] | [-97.5 to -84.9] | [-100.2 to -86.6] | [-94.5 to -87.7] | [0.5 to 24.7] | [-98.9 to -74.5] |
| Urine S-PMA | (Units) | Summary |  | | | | | | |
| Day -1 (BL) | (μg over 24h) | Mean (SD) | 5.0 (4.5) | 5.6 (3.9) | 4.3 (2.5) | 6.5 (5.6) | 5.4 (4.2) | 5.4 (3.2) | 4.6 (3.6) |
| Day 5 | (μg over 24h) | Mean (SD) | 0.2 (0.1) | 0.2 (0.1) | 0.2 (0.1) | 0.2 (0.1) | 0.2 (0.1) | 6.2 (4.1) | 0.2 (0.1) |
| Difference  (absolute) | (μg over 24h) | Mean (SD) | -4.9 (4.5) | -5.5 (3.9) | -4.1 (2.5) | -6.3 (5.6) | -5.2 (4.2) | 0.8 (1.5) | -4.3 (4.0) |
|  | (μg over 24h) | Median | -4.1 | -5.8 | -4.2 | -5.3 | -5.1 | 0.3 | -3.1 |
|  | (μg over 24h) | 95% CI | [-7.3 to -2.4] | [-7.6 to -3.3] | [-5.5 to -2.7] | [-9.4 to -3.3] | [-6.3 to -4.1] | [-0.0 to 1.7] | [-7.0 to -1.6] |
| Difference  (% reduction) | (% reduction) | Mean (SD) | -93.3 (5.8) | -93.9 (3.1) | -93.5 (2.8) | -93.9 (3.6) | -93.7 (3.9) | 19.7 (25.7) | -93.7 (4.4) |
|  | (% reduction) | Median | -95.9 | -94.7 | -93.2 | -94.9 | -94.7 | 18.4 | -95.9 |
|  | (% reduction) | 95% CI | [-96.5 to -90.1] | [-95.6 to -92.2] | [-95.1 to -92.0] | [-95.9 to -92.0] | [-94.7 to -92.6] | [5.5 to 34.0] | [-96.7 to -90.8] |
| Blood COHb | (Units) | Summary |  | | | | | | |
| Day -1 (BL) | (%) | Mean (SD) | 6.7 (1.9) | 6.8 (1.7) | 7.2 (1.3) | 7.4 (2.3) | 7.0 (1.8) | 6.7 (1.5) | 6.8 (1.7) |
| Day 5 | (%) | Mean (SD) | 1.8 (0.3) | 2.0 (0.3) | 1.8 (0.4) | 1.8 (0.2) | 1.9 (0.3) | 7.5 (1.9) | 1.9 (0.4) |
| Difference  (absolute) | (%) | Mean (SD) | -4.9 (1.9) | -4.8 (1.7) | -5.4 (1.5) | -5.6 (2.3) | -5.1 (1.9) | 0.8 (1.4) | -4.6 (1.7) |
|  | (%) | Median | -4.5 | -4.6 | -5.4 | -4.8 | -4.9 | 1.1 | -4.7 |
|  | (%) | 95% CI | [-6.0 to -3.8] | [-5.7 to -3.9] | [-6.2 to -4.5] | [-6.9 to -4.3] | [-5.6 to -4.7] | [+0.0 to 1.6] | [-5.7 to -3.4] |
| Difference  (% reduction) | (% reduction) | Mean (SD) | -71.2 (8.8) | -68.7 (8.9) | -73.8 (8.6) | -73.3 (7.7) | -71.8 (8.5) | 13.3 (22.7) | -69.1 (10.0) |
|  | (% reduction) | Median | -70.7 | -70.8 | -76.6 | -72.9 | -72.8 | 13.6 | -71.4 |
|  | (% reduction) | 95% CI | [-76.0 to -66.3] | [-73.6 to -63.8] | [-78.6 to -69.1] | [-77.5 to -69.1] | [-74.0 to -69.6] | [0.7 to 25.9] | [-75.8 to -62.4] |

* Paired Day5 - Day -1 (BL).
** Paired Day 5 vs. Day -1 (BL) Percent Reduction.
***2-Sided 95% CI
VT = Virginia Tobacco
