# Supplementary material for: Five-Day Changes in Biomarkers of Exposure Among Adult Smokers After Completely Switching From Combustible Cigarettes to a Nicotine-Salt Pod System
Source: Nicotine Tob Res. 2019 Nov 5;22(8):1285–93. doi: 10.1093/ntr/ntz206 (PMC7364828; doi:10.1093/ntr/ntz206)
Supplement: ntz206_suppl_Suplemental_Table_S6 [file ntz206_suppl_suplemental_table_s6.docx]

Table S6. Mean Urge to Smoke Usual Brand Cigarette by Cohort and Time Point (± SD)

|  | NSPS Cohorts | | | |  | Combustible Cigarette | Abstinence |
| --- | --- | --- | --- | --- | --- | --- | --- |
| Study Day | VT | Mint | Mango | Creme |  |  |  |
| Day -1 | 53.4 ± 26.50 [n = 15] | 35.3 ± 19.94 [n = 15] | 45.0 ± 22.28 [n = 15] | 47.3 ± 21.70 [n = 15] |  | 35.5 ± 20.87 [n = 15] | 49.3 ± 22.52 [n = 15] |
| Day 1 | 58.7 ± 31.00 [n = 15] | 41.5 ± 24.02 [n = 15] | 39.5 ± 27.15 [n = 15] | 44.1 ± 28.00 [n = 15] |  | 25.9 ± 20.92 [n = 15] | 76.3 ± 17.18 [n = 13] |
| Day 2 | 49.9 ± 29.89 [n = 15] | 42.3 ± 27.67 [n = 15] | 45.4 ± 29.99 [n = 15] | 38.8 ± 30.51 [n = 15] |  | 20.9 ± 19.20 [n = 15] | 78.2 ± 17.72 [n = 12] |
| Day 3 | 51.3 ± 27.48 [n = 15] | 37.0 ± 29.25 [n = 15] | 46.7 ± 32.99 [n = 15] | 36.5 ± 34.28 [n = 15] |  | 23.8 ± 22.39 [n = 15] | 72.9 ± 19.53 [n = 11] |
| Day 4 | 49.0 ± 30.24 [n = 15] | 39.0 ± 32.84 [n = 15] | 43.6 ± 30.12 [n = 15] | 33.6 ± 33.08 [n = 15] |  | 22.3 ± 23.19 [n = 15] | 60.3 ± 31.36 [n = 11] |
| Day 5 | 53.5 ± 26.87 [n = 15] | 36.7 ± 28.57 [n = 15] | 52.1 ± 29.74 [n = 15] | 37.6 ± 33.94 [n = 15] |  | 23.9 ± 24.56 [n = 15] | 55.1 ± 34.43 [n = 11] |

Note. Values are presented as arithmetic mean ± SD

0=Not at all and 100=Extreme

Subscales are calculated based on the original scales and rounded to one decimal point.

VT = Virginia Tobacco
