# Supplementary material for: Five-Day Changes in Biomarkers of Exposure Among Adult Smokers After Completely Switching From Combustible Cigarettes to a Nicotine-Salt Pod System
Source: Nicotine Tob Res. 2019 Nov 5;22(8):1285–93. doi: 10.1093/ntr/ntz206 (PMC7364828; doi:10.1093/ntr/ntz206)
Supplement: ntz206_suppl_Suplemental_Table_S7 [file ntz206_suppl_suplemental_table_s7.docx]

Table S7: Adverse Events, Severity and Relationship by Period and Cohort

| **Category** | **Number of Subjects Reporting Event** | **Number of Events Reported** | **Severity** | | | **Relationship to Product or Condition** | | | | |
| --- | --- | --- | --- | --- | --- | --- | --- | --- | --- | --- |
|  |  |  | **Mild** | **Moderate** | **Severe** | **Unrelated** | **Unlikely** | **Possibly** | **Probably** | **Likely** |
| **Baseline Period (Usual Brand Cigarette, n = 90)** | **14** | **16** | **16** | **0** | **0** | **14** | **2** | **0** | **0** | **0** |
| Agitation | 1 | 1 | 1 | 0 | 0 | 1 | 0 | 0 | 0 | 0 |
| Back pain | 2 | 2 | 2 | 0 | 0 | 2 | 0 | 0 | 0 | 0 |
| Constipation | 2 | 2 | 2 | 0 | 0 | 0 | 2 | 0 | 0 | 0 |
| Headache | 7 | 7 | 7 | 0 | 0 | 7 | 0 | 0 | 0 | 0 |
| Laceration | 1 | 1 | 1 | 0 | 0 | 1 | 0 | 0 | 0 | 0 |
| Nasal congestion | 1 | 1 | 1 | 0 | 0 | 1 | 0 | 0 | 0 | 0 |
| Vessel puncture site bruise | 1 | 1 | 1 | 0 | 0 | 1 | 0 | 0 | 0 | 0 |
| Vomiting | 1 | 1 | 1 | 0 | 0 | 1 | 0 | 0 | 0 | 0 |
| **Biomarker Assessment Period (n = 90)** | **18** | **26** | **25** | **1** | **0** | **12** | **6** | **5** | **2** | **1** |
| **NSPS Virginia Tobacco (n = 15)** | **3** | **3** | **3** | **0** | **0** | **2** | **1** | **0** | **0** | **0** |
| Abnormal dreams | 1 | 1 | 1 | 0 | 0 | 0 | 1 | 0 | 0 | 0 |
| Presyncope (vasovagal reaction with blood draw) | 2 | 2 | 2 | 0 | 0 | 2 | 0 | 0 | 0 | 0 |
| **NSPS Mint (n = 15)** | **1** | **2** | **2** | **0** | **0** | **1** | **0** | **0** | **0** | **1** |
| Oral discomfort (burning sensation - tongue and lips) | 1 | 1 | 1 | 0 | 0 | 0 | 0 | 0 | 0 | 1 |
| Skin abrasion | 1 | 1 | 1 | 0 | 0 | 1 | 0 | 0 | 0 | 0 |
| **NSPS Mango (n = 15)** | **4** | **9** | **9** | **0** | **0** | **1** | **4** | **2** | **2** | **0** |
| Burning sensation mucosal (mouth) | 1 | 1 | 1 | 0 | 0 | 0 | 0 | 0 | 1 | 0 |
| Constipation | 1 | 1 | 1 | 0 | 0 | 0 | 1 | 0 | 0 | 0 |
| Cough | 1 | 1 | 1 | 0 | 0 | 0 | 0 | 1 | 0 | 0 |
| Diarrhea | 1 | 1 | 1 | 0 | 0 | 0 | 1 | 0 | 0 | 0 |
| Dyspepsia | 2 | 2 | 2 | 0 | 0 | 0 | 2 | 0 | 0 | 0 |
| Hypoesthesia oral (numbness in the mouth) | 1 | 1 | 1 | 0 | 0 | 0 | 0 | 0 | 1 | 0 |
| Oropharyngeal pain (sore throat) | 1 | 1 | 1 | 0 | 0 | 0 | 0 | 1 | 0 | 0 |
| Vessel puncture site pain | 1 | 1 | 1 | 0 | 0 | 1 | 0 | 0 | 0 | 0 |
| **NSPS Creme (n = 15)** | **4** | **6** | **6** | **0** | **0** | **2** | **1** | **3** | **0** | **0** |
| Constipation | 1 | 1 | 1 | 0 | 0 | 0 | 1 | 0 | 0 | 0 |
| Cough | 2 | 2 | 2 | 0 | 0 | 0 | 0 | 2 | 0 | 0 |
| Oropharyngeal pain (sore throat) | 1 | 1 | 1 | 0 | 0 | 0 | 0 | 1 | 0 | 0 |
| Vessel puncture site hemorrhage | 1 | 2 | 2 | 0 | 0 | 2 | 0 | 0 | 0 | 0 |
| **Usual Brand Cigarette (n = 15)** | **3** | **3** | **3** | **0** | **0** | **3** | **0** | **0** | **0** | **0** |
| Constipation | 1 | 1 | 1 | 0 | 0 | 1 | 0 | 0 | 0 | 0 |
| Skin abrasion | 1 | 1 | 1 | 0 | 0 | 1 | 0 | 0 | 0 | 0 |
| Vessel puncture site pain | 1 | 1 | 1 | 0 | 0 | 1 | 0 | 0 | 0 | 0 |
| **Smoking Abstinence (n = 15)** | **3** | **3** | **2** | **1** | **0** | **3** | **0** | **0** | **0** | **0** |
| Back pain | 1 | 1 | 1 | 0 | 0 | 1 | 0 | 0 | 0 | 0 |
| Irritability | 1 | 1 | 0 | 1 | 0 | 1 | 0 | 0 | 0 | 0 |
| Localized edema | 1 | 1 | 1 | 0 | 0 | 1 | 0 | 0 | 0 | 0 |
