# Supplementary figures and images for: Five-Day Changes in Biomarkers of Exposure Among Adult Smokers After Completely Switching From Combustible Cigarettes to a Nicotine-Salt Pod System
Source: Nicotine Tob Res. 2019 Nov 5;22(8):1285–93. doi: 10.1093/ntr/ntz206 (PMC7364828; doi:10.1093/ntr/ntz206)

Figure S1. Change in urge to smoke.

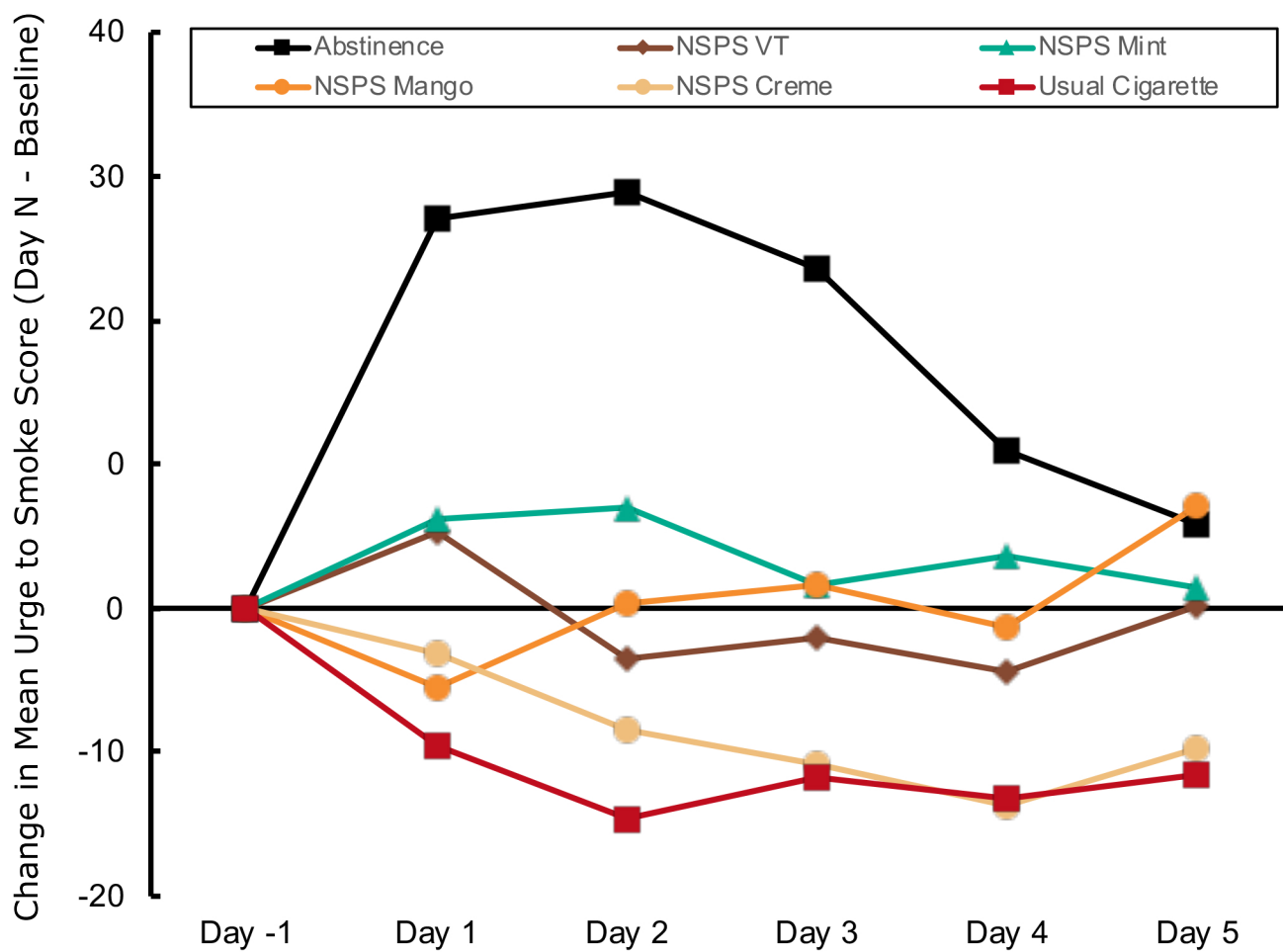

VT = Virginia Tobacco

Supplement: ntz206_suppl_Suplemental_Figure_1 [file ntz206_suppl_suplemental_figure_1.pdf]
