# Supplementary material for: Five-Day Changes in Biomarkers of Exposure Among Adult Smokers After Completely Switching From Combustible Cigarettes to a Nicotine-Salt Pod System
Source: Nicotine Tob Res. 2019 Nov 5;22(8):1285–93. doi: 10.1093/ntr/ntz206 (PMC7364828; doi:10.1093/ntr/ntz206)
Supplement: ntz206_suppl_Suplemental_Figure_2 [file ntz206_suppl_suplemental_figure_2.pdf]

Figure S2: WISDM primary (PDM) and secondary (SDM) dependence motives mean scores at baseline and day 5.

WISDM (PDM) - Baseline vs. Day 5

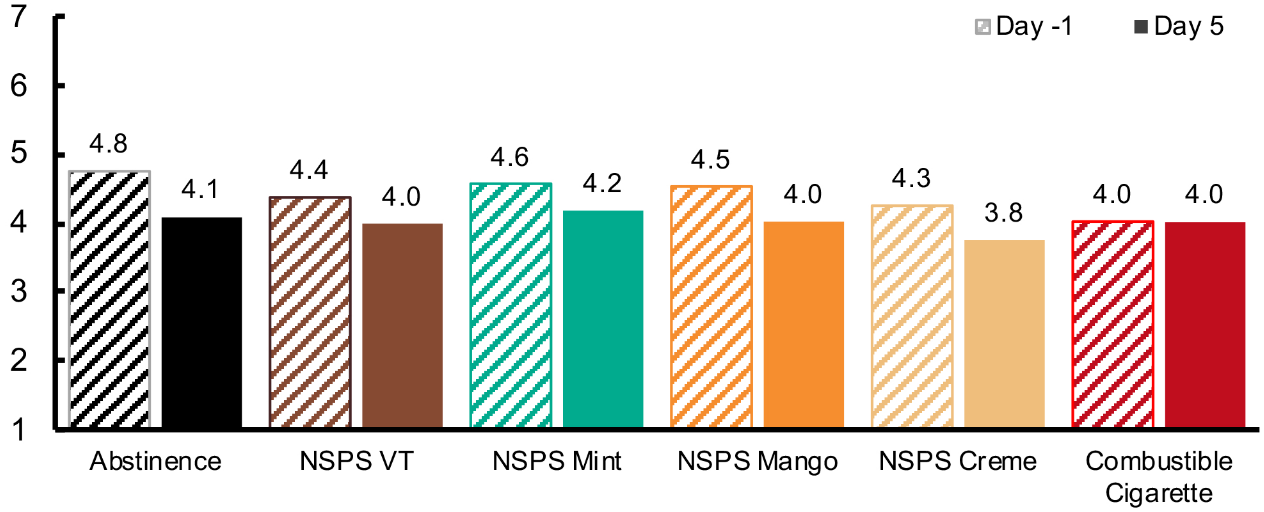

WISDM (SDM) - Baseline vs. Day 5

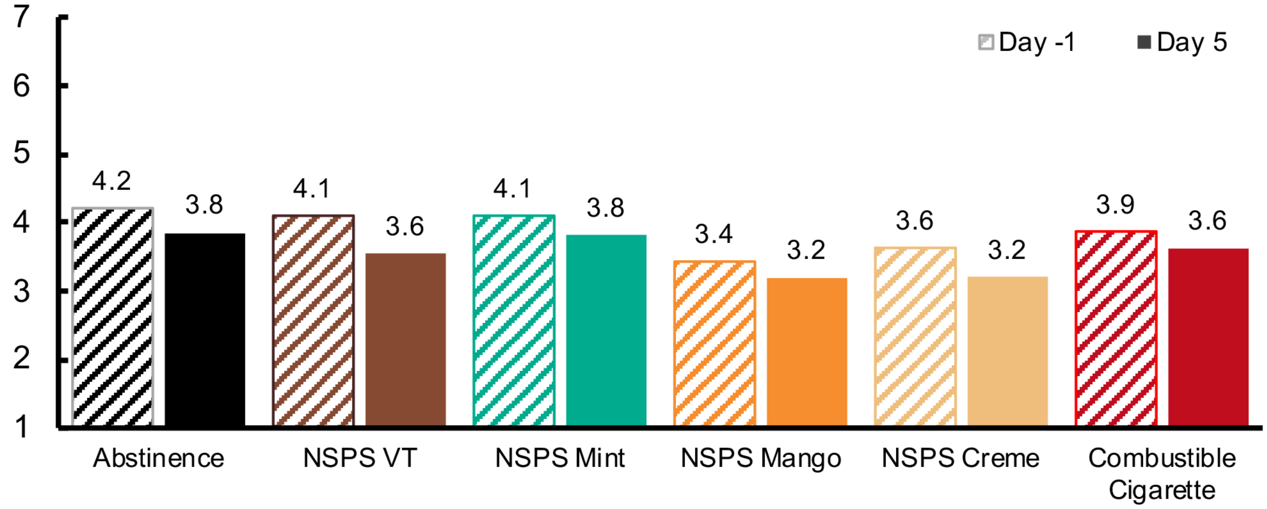

VT = Virginia Tobacco
